# Self-determination in Physical Exercise Predicts Creative Personality of College Students: The Moderating Role of Positive Affect

**DOI:** 10.3389/fspor.2022.926243

**Published:** 2022-07-11

**Authors:** Shaoqing Chen, Qing Wang, Xinya Wang, Liying Huang, Dongdong Zhang, Baoguo Shi

**Affiliations:** ^1^Physical Education Teaching and Research Department, Capital Normal University, Beijing, China; ^2^Beijing Key Laboratory of Learning and Cognition, School of Psychology, Capital Normal University, Beijing, China; ^3^School of Teacher Education, Capital Normal University, Beijing, China

**Keywords:** physical exercise, self-determination, creative personality, positive affect, college student

## Abstract

Although previous studies indicated that intrinsic motivation and positive affect were important for creativity and proved the benefits of physical exercise for creativity, little is known about underlying this relationship between self-determination in physical exercise and creative personality among college students. Based on prior studies and theoretical models, the current study investigated the relationship between self-determination in physical exercise and creativity and the moderating role of positive affect in the relationship between self-determination in physical exercise and creative personality. This model was analyzed with 1,201 Chinese college students (Mean age = 20.10 years, SD = 0.93). Participants filled out the physical exercise self-determination scale, the Williams creativity assessment packet, and the satisfaction with life scale. The results indicated that self-determination in physical exercise was significantly positively correlated with the creative personality of college students, including risk-taking, curiosity, challenge, and imagination. Moreover, self-determination in physical exercise could significantly positively predict the creative personality of college students. Moderation analysis further showed that the relationship between self-determination in physical exercise and creative personality was robust for college students with low levels of positive affect. These findings suggest the importance of cultivating students' creative ability through improving exercise autonomy, especially for those college students with a low level of positive affect.

## Introduction

Creativity, characterized as the generation of novel and useful ideas by individuals, has been integrated into daily life and links with various domains such as science, art, and sports (Amabile, 1982, 1983; Craft, 2006). Moreover, it is critical for the competition in different nations and the development of individuals (Gan et al., 2020). According to the 4P model of creativity, creativity is defined by the person, product, process, and place; among them, creative personality is an essential component of creativity (Rhodes, 1961; Kozbelt et al., 2010). Creative personality refers to the sum of various psychological qualities of an individual with a tendency for creative activities and is the intrinsic basis of creativity (Kozbelt et al., 2010). Specifically, creative personality includes risk-taking, curiosity, challenge, and imagination (Williams, 1980). There is extensive evidence that creativity is associated with physical exercise (Steinberg et al., 1997; Frith and Loprinzi, 2018; Román et al., 2018; Zhao et al., 2022). Significantly, prior studies indicated that individuals participate in activities out of extrinsic motivation, such as self-improvement, and it is conducive to the increase of creativity (Amabile, 1985; Hennessey and Amabile, 1998; Sansone and Harackiewicz, 2000; Moneta and Siu, 2002; Gerhart and Fang, 2015). By that analogy, individuals' self-determination in physical exercise can also improve their creativity. The dynamic componential model of creativity points out that intrinsic motivation, which is individuals taking part in an activity because it is enjoyable or meaningful, was the necessary ingredient for creativity (Amabile, 1983, 1988; Amabile and Pratt, 2016). Moreover, the positive affect plays a crucial role in the creativity-related process (Amabile and Pratt, 2016). Given the effects of physical exercise, motivation, and emotion on creative personality, it is of theoretical and practical importance to explore those factors related to creative personality.

However, to date, most research only focuses on the independent effects of physical exercise, motivation, and positive emotions on creativity. Few researchers have paid attention to the influence of self-determination in physical exercise on the creative personality and the moderating mechanism of positive affect between self-determination in physical exercise and creative personality. Due to the need for creativity and the potential benefits of physical exercise, it is important to explore the impact of combining physical exercise with autonomous motivation on creative personality and investigate its internal affect moderating mechanism.

### Self-determination in Physical Exercise and Creative Personality

Self-determination in physical exercise refers to the subjective feeling of the degree of the intrinsic drive of an individual during physical exercise (Wang et al., 2019). Specifically, self-determination in physical exercise includes a sense of belonging, identification, internal integration, competence in physical exercise, and body confidence (Fang et al., 2012). Individuals with a high level of self-determination in physical exercise are more willing to engage voluntarily and actively (Deci and Ryan, 2004, 2008; Seymour et al., 2021), so self-determination in physical exercise is the autonomous motivation to motivate individuals to exercise. According to self-determination theory, autonomous motivation includes identified regulation, integrated regulation, and intrinsic motivation. The higher the intrinsic motivational orientation, the higher the concentration, persistence, and willingness to exert effort (Deci and Ryan, 1985). Specific to creativity, the influence of motivation on creativity is achieved through the integration of the individual's intrinsic and extrinsic motivations and environment (Hao and Tang, 2017). Therefore, motivation combined with specific activities, such as physical exercise, is more beneficial and enhances creativity. Moreover, autonomous motivation provides more freedom than controlled motivation, allowing individuals to think more broadly and promoting creativity (Eisenberger and Shanock, 2003). Thus, self-determination in physical exercise may relate to one's creativity.

In addition, from the perspective of individual cognitive development and information processing, creativity depends to some extent on the development and maturity of cranial nerves, including the cognitive processing such as information processing, control, and monitoring, and requires the coordination of the perceptual system, memory, thinking, and speech to perform the corresponding creative functions (Urban, 1991; Feldman, 1999). Self-determination enables individuals actively and frequently to participate in physical exercise (Seymour et al., 2021). Physical exercise changes the level of activation of brain regions such as the anterior cingulate gyrus and frontal lobe, ultimately enhancing cognitive abilities such as central executive function (Xia et al., 2018). Thus, self-determination in physical exercise may have a promoting effect on creative performance.

Specific to the relationship between self-determination in physical exercise and creative personality, previous studies pointed out that physical exercise was linked with an individual's personality (Rhodes, 2006; Rhodes and Pfaeffli, 2012; Wilson and Dishman, 2015; Sutin et al., 2016; Buhaş and Stance, 2017). Compared to individuals with less physical activity, individuals with more physical activity have significantly higher extroversion, neuroticism, conscientiousness, and openness (Wilson and Dishman, 2015). Prior studies also showed that extraversion and conscientiousness were both associated with more intrinsic regulation of exercise behaviors and less external regulation (Ingledew et al., 2004). These results indicated a close relationship between the intrinsic motivation of sports and individual personality characteristics. Moreover, the theory of integrating traits indicated that traits were tools for satisfying basic psychological needs of self-determination; basic psychological needs of self-determination can (partially) explain traits (Prentice et al., 2019). Given that creative personality is the intrinsic basic of creativity and one of the personality traits, and self-determination is the autonomous driving force to meet the needs of physical exercise, it is reasonable to deduce that the self-determination in physical exercise could predict individual's creative personality. However, few empirical studies have directly explored the relationship between self-determination in physical exercise and creative personality. Based on the above theories, we point out the following hypothesis:

**Hypothesis 1**: Self-determination in physical exercise could positively predict creative personality.

### Moderating Role of Positive Affect

Although self-determination in physical exercise was related to creative personality, it is unlikely that all individuals are influenced by this effect equally. Therefore, it is necessary to identify the potential moderators in the relationship between self-determination in physical exercise and creative personality. Despite the scarce empirical studies, it is reasonable that positive affect can be one of the moderators in the relationship between self-determination in physical exercise and creative personality.

The extent to which an individual has positive affect may also influence the relationship between self-determination in physical exercise and creative personality. The “broaden-and-build” theory of positive affect posits that positive affect broadens people's momentary reserve of thinking activities, and helps individuals build their lasting personal resources (Zhang et al., 2017), thereby increasing individuals' creativity. Moreover, previous studies found that the positive affect moderated the relationship between managerial creativity and organizational performance; creativity in management could positively predict organizational performance when individuals experienced more positive affect (Yu et al., 2021). Nevertheless, when creative personality is associated with motivation, the moderating direction of emotion may be changed. A study pointed out that low positive affect in approach motivation enhances memory for peripherally presented information. Higher positive affect hinders the processing of general information (Gable and Harmon-Jones, 2010b). In other words, individuals with a lower level of positive affect are more likely to transform motivation (e.g., physical exercise self-determination) into the exploration of creative activities (e.g., risk-taking and curiosity). Thus, it is theoretically logical to assume that positive affect can moderate the relationships between self-determination in physical exercise and creative personality. However, no studies have examined the moderating effect of positive affect in the relationships between self-determination in physical exercise and creative personality. Based on the literature discussed above, the following hypothesis was established:

**Hypothesis 2**: Positive affect would moderate the relationships between self-determination in physical exercise and creative personality.

### The Present Study

In summary, the purposes of the study were as follows. First, this study tested whether self-determination in physical exercise would positively affect college students' creative personality. Second, the study explored whether positive affect would moderate the relationship between self-determination in physical exercise and creative personality.

## Materials and Methods

### Participants

**Figure 1:**
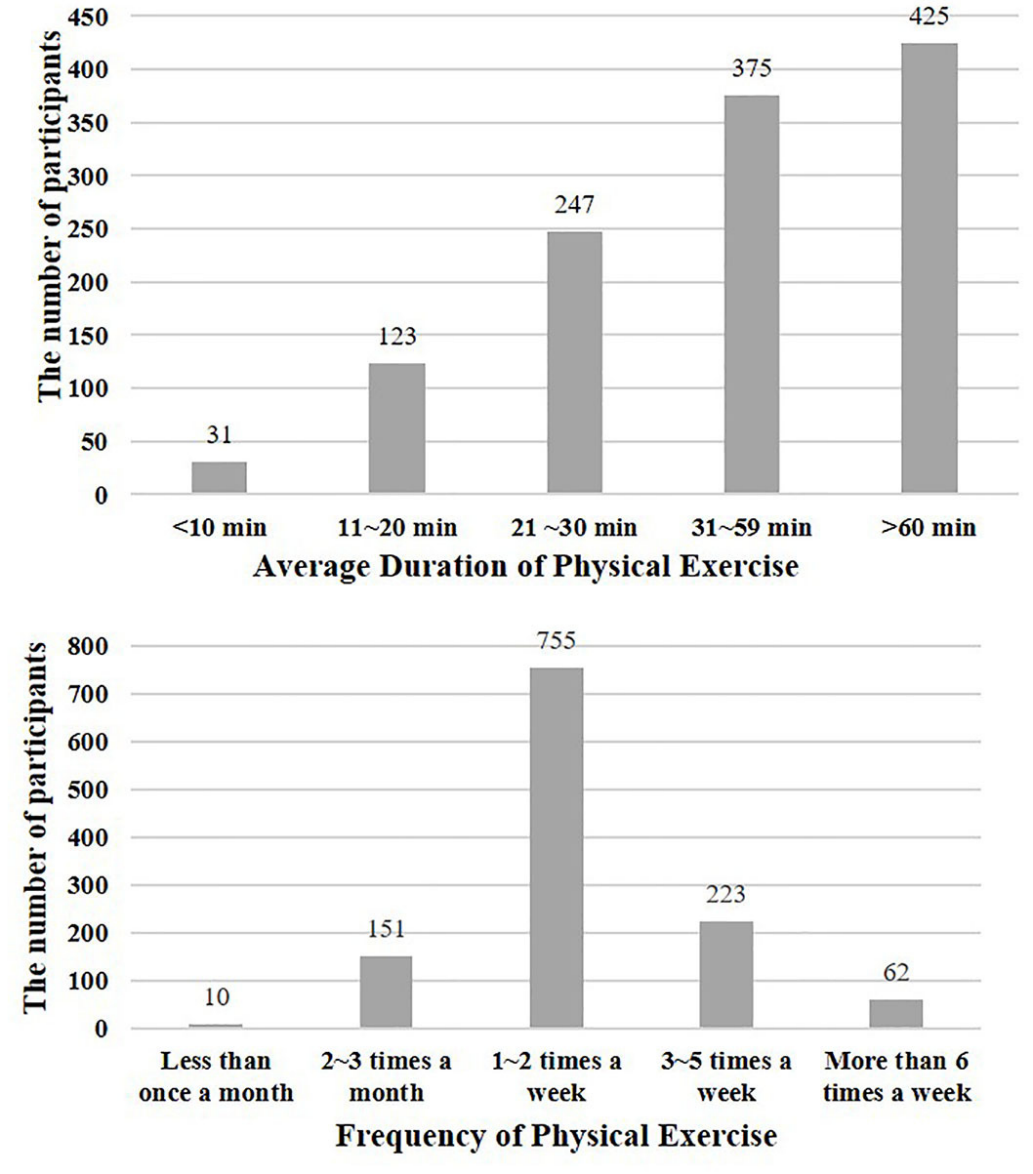
The descriptive statistics of college students' physical exercise.

We recruited 1,201 college students (73.11% of the participants were female) from the capital normal university in Beijing, China. The average age of the participants was 20.10 years (*SD*_age_ = 0.93, range = 17–27 years), with 59.03% of the students were freshmen, and the rest were sophomore. The survey includes demographic variables, physical exercise self-determination questionnaires, Williams Creativity Assessment Packet, and the positive affect frequency scale. In addition, we investigated the frequency and average duration of participants' physical activities. The results were presented in Figure 1.

### Measures

#### The Physical Exercise Self-determination Scale

The self-determination in physical exercise was measured by the Chinese version of the Physical Exercise Self-Determination Scale (Fang et al., 2012). The scale consists of 16 items that cover four dimensions: the sense of belonging and identification (e.g., “Physical exercise is good for my coursework”), body self-confidence (e.g., “My fit body is attractive during physical exercise”), sense of competence (e.g., “I have good sports knowledge and skills”), and sense of internal integration (e.g., “Physical exercise is my hobby, and I enjoy it”). The last item was reverse scored. Each item was rated on a 5- point Likert scale from 1 (strongly disagree) to 5 (strongly agree), with higher scores indicating higher levels of self-determination in physical exercise. This scale was used in previous studies (Wang et al., 2019). The scale has good reliability (the Cronbach's α is 0.937) and validity, with the fit indices of modified model is acceptable (RMSEA = 0.069 < 0.08, CFI = 0.965 > 0.90, and SRMR = 0.042 < 0.08).

### The Positive and Negative Affect Schedule (PANAS)

The positive affect was measured by the Chinese version of Positive and Negative Affect Schedule (PANAS) (Yan et al., 2002) revised based on based on the original version (Watson et al., 1988; Diener et al., 1995; Huebner and Dew, 1995). The scale consists of 14 items covering positive affect and negative affect. We use the dimensions of positive affect to measure one's positive affective. The scale consists of 6 words describing positive affect (e.g., “pleasure,” “happiness,” and “Proud”), and the participants were asked to answer the frequency of these types of affects that they experienced most of the time. Each item was rated on a 7- point Likert scale from 1 (never) to 7 (always), with higher scores indicating higher levels of the positive affect. The scale has good reliability (the Cronbach's α is 0.871) and validity, with the fit indices of modified model is acceptable (RMSEA = 0.073 < 0.08, CFI = 0. 965 > 0.90, and SRMR = 0.074 < 0.08).

### Williams Creativity Assessment Packet

The creativity was measured by the Chinese version of Williams creativity assessment packet (Lin and Wang, 1997) revised based on the original version (Williams, 1980). The scale consists of 50 items that cover four dimensions: risk-taking (e.g., “At school, I like to try to make guesses about things or questions, even if I do not necessarily guess them right, it does not matter”), curiosity (e.g., “I like to look carefully at things I have not seen before to learn more about them”), imagination (e.g., “I like to imagine things I want to know or do”), and challenge (e.g., “I like varied and imaginative stories”). Each item was rated on a 3- point Likert scale from 1 (complete non-conformity) to 3 (complete conformity), with a higher score indicating higher levels of the risk-taking, curiosity, imagination, challenge and creative personality. The scale has good reliability (the Cronbach's α is 0.902) and validity, with the fit indices of modified model is acceptable (RMSEA = 0.040 < 0.08, CFI = 0. 901 > 0.90, and SRMR = 0.047 < 0.08).

### Procedure

This study provided a web-based survey platform for participants to voluntarily fill out the anonymous' survey and spent approximately about 5–10 min. The Ethics in Human Research Committee of the first author's university approved all materials and procedures. We also informed participants of the purpose of the study and the principles of voluntary participation and withdrew from the survey. Finally, we obtained informed consent from the participants.

### Data Analysis

First, the descriptive statistics, correlation analysis, and regression analysis were conducted using SPSS 25.0. Second, we conducted model 1 of the PROCESS macro 3.3 for SPSS to determine the moderating effects of the positive affect between self-determination in physical exercise and creativity (Hayes, 2017). Third, we performed confirmatory factor analysis on the scales and tested the fitting index of the moderation model by Mplus 8.0. The models were acceptable if RMSEA < 0.08, the CFI > 0.90, and the SRMR < 0.08 (Bentler, 1990; Browne and Cudeck, 1992; Hu and Bentler, 1999). Finally, we used the SPSS macro “Interaction and simple slopes test with two continuous variables” to generate the plots and simple slopes analyses (Hayes, 2017).

## Results

### Common Method Bias Analysis

This study used self-reported data, so there may be a common method bias problem (Siemsen et al., 2010). This study used reverse scoring of some items to control common method bias. Then, Harman's single-factor test showed that 14 factors with characteristic roots greater than one were extracted from the unrotated exploratory factor analysis results, and the maximum factor variance explanation rate was 20.7% (<40%) which met the test criteria. Therefore, there was no severe common method bias in this study.

### Preliminary Analyses

The descriptive statistics and correlation analysis for all study variables are presented in Table 1. The results showed that college students with a high level of self-determination in physical exercise were more likely to have a high level of creative personality (*r* = 0.28, *p* < 0.001), risk-taking, curiosity, imagination, and challenge (*r* = 0.13–0.34, *p* < 0.001). Besides, college students with high level of positive affect were more likely to have high level of creative personality (*r* = 0.33, *p* < 0.001), risk-taking, curiosity, imagination, challenge (*r* = 0.17–0.39, *p* < 0.001) and self-determination in physical exercise (*r* = 0.46, *p* < 0.001). In addition, college students with a high level of creative personality were more likely to have a high sense of belonging and identification, sense of competence, and body self-confidence (*r* = 0.20–0.31, *p* < 0.001).

**Table 1:** Means, standard deviations, and Pearson-correlations among variables.

**Variable**	***M***	***SD***	**1**	**2**	**3**	**4**	**5**	**6**	**7**	**8**	**9**	**10**	**11**
1. Creative personality	1.80	0.24	1										
2. Risk-taking	1.83	0.26	0.87^***^	1									
3. Curiosity	1.75	0.29	0.91^***^	0.74^***^	1								
4. Imagination	1.93	0.30	0.82^***^	0.60^***^	0.62^***^	1							
5. Challenge	1.71	0.27	0.85^***^	0.69^***^	0.73^***^	0.53^***^	1						
6. Positive affect	3.23	1.09	0.33^***^	0.39^***^	0.31^***^	0.17^***^	0.29^***^	1					
7. SDPE	2.28	0.74	0.28^***^	0.34^***^	0.29^***^	0.13^***^	0.24^***^	0.46^***^	1				
8. SBI	1.78	0.70	0.31^***^	0.33^***^	0.31^***^	0.16^***^	0.29^***^	0.40^***^	0.87^***^	1			
9. BSC	2.54	1.06	0.20^***^	0.27^***^	0.19^***^	0.11^***^	0.13^***^	0.41^***^	0.85^***^	0.60^***^	1		
10. SC	2.36	0.85	0.23^***^	0.29^***^	0.25^***^	0.12^***^	0.17^***^	0.43^***^	0.90^***^	0.65^***^	0.76^***^	1	
11. SII	2.44	0.83	0.20^***^	0.24^***^	0.22^***^	0.04	0.21^***^	0.33^***^	0.80^***^	0.62^***^	0.54^***^	0.67^***^	1

*N = 1,201; SDPE, Self-determination in physical exercise; SBI, Sense of belonging and identification; BSC, Body self-confidence; SC, Sense of competence; SII, Sense of internal integration;*

*** *Represent p < 0.001*.

### The Predictive Effect of Self-determination in Physical Exercise on Creative Personality

In Hypothesis 2, the study expected that self-determination in physical exercise would predict creative personality. Regression analysis was conducted using SPSS 25.0 to test the predictive effect of self-determination in physical exercise on creative personality. As shown in Table 2, after controlling for gender and grade, the regression analysis results showed that self-determination in physical exercise significantly positively predicted creative personality (*β* = 0.285, *p* < 0.001). In addition, self-determination in physical exercise significantly positively predicted the risk-taking (*β* = 0.340, *p* < 0.001), curiosity (*β* = 0.289, *p* < 0.001), challenge (*β* = 0.239, *p* < 0.001), and imagination (*β* = 0.134, *p* < 0.001) separately. Thus, Hypothesis 1 was supported.

**Table 2:** Hierarchical regression analysis of creative personality and its dimensions on self-determination in physical exercise.

**Dependent variable**	**Model**	**Predictor variable**	***β***	***t***	***ΔR^**2**^***	***F***
Creative personality	1	Gender	−0.008	−0.270	0.000	0.113
		Grade	0.011	0.390		
	2	SDPE	0.285	10.255^***^	0.081	105.174^***^
Risk-taking	1	Gender	−0.027	−0.991	0.000	0.009
		Grade	0.007	0.260		
	2	SDPE	0.340	12.454^***^	0.115	155.105^***^
Curiosity	1	Gender	0.006	0.221	0.001	0.49
		Grade	0.001	0.053		
	2	SDPE	0.289	10.407^***^	0.083	108.296^***^
Imagination	1	Gender	−0.005	−0.175	0.000	0.042
		Grade	−0.003	−0.091		
	2	SDPE	0.134	4.653^***^	0.018	21.651^***^
Challenge	1	Gender	−0.006	−0.201	0.001	0.565
		Grade	0.036	1.273		
	2	SDPE	0.239	8.496^***^	0.057	72.176^***^

*N = 1,201; SDPE, Self-determination in physical exercise;*

*** *Represent p < 0.001*.

### The Moderating Role of Positive Affect

In Hypothesis 2, the current study assumed that positive affect would moderate the relationship between self-determination in physical exercise and creative personality. To examine the moderated hypothesis, we used Model 1 in the SPSS macro program PROCESS developed by Hayes to examine the moderating effect (Hayes, 2017). Moreover, we further construct moderation model in latent variables, and the results showed that the fitting index of the modified moderation model is acceptable (RMSEA = 0.078 < 0.08, CFI = 0. 961 > 0.90 and SRMR = 0.043 < 0.08). As presented in Table 3, the moderation results showed that positive affect moderated the link between self-determination in physical exercise and creative personality (*β* = −0.088, *p* < 0.001). In addition, we further tested the moderating effect of positive affect on the relationship between self-determination in physical exercise and risk-taking, curiosity, challenge, and imagination. The results showed that positive affect separately moderated the relationship between self-determination physical exercise and risk-taking (*β* = −0.079, *p* < 0.01), curiosity (*β* = −0.095, *p* < 0. 001), challenge (*β* = −0.066, *p* < 0.05), and imagination (*β* = −0.058, *p* < 0.05).

**Table 3:** Regression analysis results of the moderating effect of positive affect.

**Variable**	**Creative personality**		
	***β***	***SE***	***t***
SDPE	0.179	0.03	5.87^***^
Positive affect	0.235	0.031	7.677^***^
SDPE × Positive affect	−0.088	0.025	−3.456^***^
*R^2^*		0.138	
*F*		*F*_(3,1197)_ = 64.018^***^	

*N = 1,201; SDPE, Self-determination in physical exercise;*

*** *Represent p < 0.001*.

**Figure 2:**
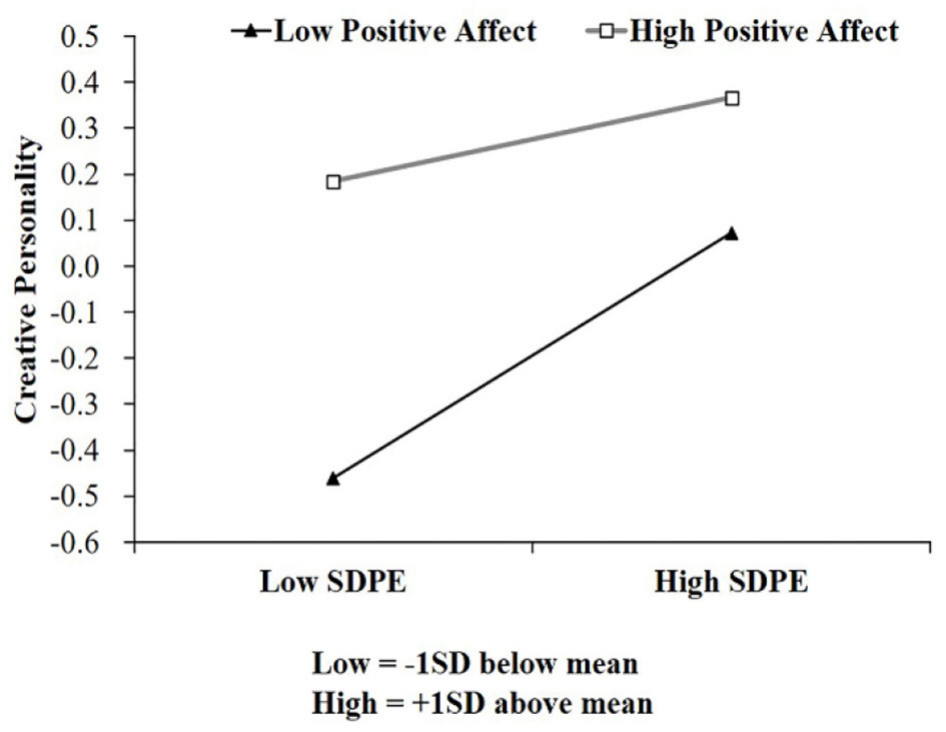
The relationship between self-determination in physical exercise and creative personality for high and low levels of positive affect. *N* = 1,201, SDPE, Self-determination in physical exercise.

Furthermore, we conducted a simple slope analysis on the link between self-determination in physical exercise and creative personality at two levels of positive affect (Table 4). According to Table 4, we plotted predicted self-determination in physical exercise against creative personality (Figure 2) separately for low and high levels of positive affect (1 SD below the mean and 1 SD above the mean, respectively). Simple slope tests indicated that for low positive affect individuals, higher levels of self-determination in physical exercise were associated with higher creative personality individuals (*b*_simple_ = 0.266, *p* < 0.001). However, for high positive affect individuals, the relationship between self-determination in physical exercise and creative personality was weakened (*b*_simple_ = 0.091, *p* < 0.05). Thus, Hypothesis 2 was supported.

**Table 4:** Simple slope analysis of interaction between self-determination in physical exercise and positive affect.

**Conditional effect of PA**	***Estimate***	***SE***	***t***	**95%**	**CI**
Mean	0.179	0.030	5.870^***^	0.119	0.238
High level (+1 SD)	0.091	0.038	2.420^*^	0.017	0.165
Low level (−1 SD)	0.266	0.041	6.420^***^	0.185	0.348

*N = 1,201; PA, positive affect*,

* *Represent p < 0.05;*

*** *Represent p < 0.001*.

## Discussion

Little research focuses on the relationship between self-determination in physical exercise and creative personality, and the moderating mechanisms underlying this relationship are still largely unknown. To fill this gap, this study used the regression analysis to investigate the relationship between self-determination in physical exercise and creative personality and the moderating effect of positive affect on the links between self-determination in physical exercise and creative personality. The results revealed that physical exercise self-determination could positively predict creative personality, such as risk-taking, curiosity, challenge, and imagination. Positive affect moderated the relationship between self-determination in physical exercise and creative personality. We will discuss each of the findings in the following sections.

### Self-determination in Physical Exercise and Creative Personality

The current study found that self-determination in physical exercise positively predicts creative personality, including risk-taking, curiosity, challenge, and imagination. To our knowledge, intrinsic motivation is an essential source of self-determination, and it plays a positive role in promoting creativity (Amabile et al., 1994; Hennessey, 2000; Eisenberger and Shanock, 2003). In sports, studies have confirmed that physical exercise self-determination, as intrinsic motivation, has a positive predictive effect on physical exercise behavior (Xiang, 2013). In addition, compared to individuals with less physical exercise, individuals with more physical exercise have higher extroversion, openness, and conscientiousness (Wilson and Dishman, 2015). The personality traits of extroversion and openness contribute to risk-taking, curiosity, challenge, and imagination (Ellis and Meneely, 2015; Li et al., 2015). From the perspective of sports, exercisers are more likely to try new things and new ways to challenge and surpass themselves and thus enhance themselves physically, intellectually, and effectively, which has an impact on creative personality, such as risk-taking, challenge, and curiosity (Bailey and Morley, 2006).

According to the basic psychological need theory, individuals have three basic psychological needs: autonomy, competence, and relatedness (Deci and Ryan, 2000, 2004). Physical exercise could achieve autonomous participation, role competence, and a sense of collective belonging. After meeting individuals' psychological needs, individuals will pursue higher needs, which can become a driving force for creativity. Therefore, it is logical that the relationship between the performance of creative personality and self-determination in physical exercise.

Moreover, the sense of belonging and identification, internal integration, body confidence, and competence in physical exercise also could positively predict creative personality. The sense of belonging and identification is an individual's conscious evaluation of behavioral goals or rules (Fang et al., 2012). When an individual comprehensively evaluation the importance of the behavior to himself or herself and can identify these rules, helping build the individual's creative personality. Competency refers to an individual's belief in his or her ability to perform the behavior competently (Fang et al., 2012). Individuals with solid competence and body confidence show a high sense of self-efficacy, so they have a stronger creative personality, such as curiosity and risk-taking (Karwowski, 2012, 2016). Internal integration refers to the behavior caused by the pleasure and satisfaction generated by the exercise activity itself, without the involvement of external conditions and complete assimilation of the rules identified by the self (Fang et al., 2012). As we mentioned above, intrinsic motivation and creativity are closely related, so individuals with a high level of internal tend to have a higher level of creative personality.

Previous research has focused on physical exercise activity or health status on creativity. Few studies have examined the influence of physical exercise self-determination on creativity and the moderating effect of positive affect. This study makes a unique contribution to dependent and independent variable indicators, which can provide empirical evidence for studies related to physical education and creativity development.

### The Moderating Role of Positive Affect

This study showed that positive affect had a negative moderating effect on the relationship between self-determination in physical exercise and creative personality. Specifically, compared to individuals with having more positive emotions, the relationship between self-determination in physical exercise and creative personality was stronger with individuals having less positive emotions. These results may be attributed to the following reasons.

The affective-motivational dimension model holds that lower positive affect may be more likely to integrate various environmental cues and exhibit exploratory behaviors and activities. In comparison, higher positive affect reduces the breadth of attention. It hinders the brain from receiving peripheral information, thereby influencing the understanding of the whole and thus decreasing the level of cognition (Lu et al., 2002; Gable and Harmon-Jones, 2008, 2010a). This suggests that the effect of positive affect on creativity may be complex (Fernández-Abascal and Díaz, 2013). A prior study showed that low survival motivational affect could improve creativity, and it may be that the processing of affect requires the consumption of attentional resources (Pessoa and Engelmann, 2010). In terms of physical activities, in collective ball games, calm and collected athletes are often better at spotting the weaknesses of their opponents, making objective and rational judgments and decisions, and promptly adjusting their strategies to defeat their opponents. This suggests that lower positive emotions may be more conducive to improving creativity through exercise.

### Implications and Limitations

The findings of the study have several important theoretical and practical implications. First, from a theoretical perspective, these findings provide insights into how self-determination physical in exercise was related to college students' creative personality, enriching and broadening people's understanding of self-determination theory and the literature on creativity. Given that self-determination and physical exercise are both powerful driving forces of individuals' creativity, it is essential to understand the self-determination in physical exercise's relationship with people's creative personality. Second, the findings showed that positive affect influenced the relationship between self-determination in physical exercise and creative personality. It enlightens us that the relationship between self-determination in physical exercise and creative personality is not constant and will be affected by individual emotions. Although positive emotion has been proved to be conducive to creativity in most previous studies, its role may be more complex and flexible when it acts as a moderator variable. Third, the present study might be helpful for the educator to increase students' intrinsic motivation to participate in physical exercise and cultivate students' creativity from the perspective of physical exercise, which realizes the improvement of college students' health and creativity.

### Limitations and Future Directions

This study has some limitations, which need to be further improved in the future. First, the sample was collected from only one college in Beijing, so the sample's representativeness needs to be improved. Future research could use diverse samples to confirm these results. Second, this study was a cross-sectional design, so the causal or bidirectional relationship between creative personality and physical exercise was unable to be verified. Future research could use the longitudinal research design to examine further the effect of self-determination in physical exercise on creative personality. Third, the mechanism of influence on the relationship between self-determination in physical exercise and creative personality is not clear. Future research could collect more variables to examine how self-determination in exercise was related to creative personality.

## Conclusion

This study indicates that self-determination in physical exercise can be a protective factor in college students' creative personality. Moreover, positive affect has a negative moderating effect on the relationship between self-determination in physical exercise and college students' creative personality. Specifically, a low level of positive affect could be a favorable factor in influencing the relationship between self-determination in physical exercise and creative personality.

## Data Availability Statement

The original contributions presented in the study are included in the article/supplementary material, further inquiries can be directed to the corresponding author.

## Ethics Statement

The studies involving human participants were reviewed and approved by the Ethics in Human Research Committee of the first author's university. The patients/participants provided their written informed consent to participate in this study.

## Author Contributions

SC and QW designed the study, analyzed the data, and wrote the manuscript. XW, LH, and DZ performed the investigation and analyzed the data. BS analyzed the data. All authors contributed to the article and approved the submitted version.

## Funding

This work was supported by the Beijing Social Science Foundation, Key Project of Social Science Plan of Beijing Municipal Commission of Education (SZ202210028016).

## Conflict of Interest

The authors declare that the research was conducted in the absence of any commercial or financial relationships that could be construed as a potential conflict of interest.

## Publisher's Note

All claims expressed in this article are solely those of the authors and do not necessarily represent those of their affiliated organizations, or those of the publisher, the editors and the reviewers. Any product that may be evaluated in this article, or claim that may be made by its manufacturer, is not guaranteed or endorsed by the publisher.

## References

[B1] Amabile T. M. (1982). Social psychology of creativity: a consensual assessment technique. J. Pers. Soc. Psychol. 43, 997–1013. 10.1037/0022-3514.43.5.99716060417

[B2] Amabile T. M. (1983). The social psychology of creativity: a componential conceptualization. J. Pers. Soc. Psychol. 45, 357–377. 10.1037/0022-3514.45.2.357

[B3] Amabile T. M. (1985). Motivation and creativity: effects of motivational orientation on creative writers. J. Pers. Soc. Psychol. 48, 393. 10.1037/0022-3514.48.2.393

[B4] Amabile T. M. (1988). A model of creativity and innovation in organizations. Res. Organ. Behav. 10, 123–167.

[B5] Amabile T. M. Hill K. G. Hennessey B. A. Tighe E. M. (1994). The Work Preference Inventory: assessing intrinsic and extrinsic motivational orientations. J. Pers. Soc. Psychol. 66, 950. 10.1037/0022-3514.66.5.950 8014837

[B6] Amabile T. M. Pratt M. G. (2016). The dynamic componential model of creativity and innovation in organizations: making progress, making meaning. Res. Organ. Behav. 36, 157–183. 10.1016/j.riob.2016.10.001

[B7] Bailey R. Morley D. (2006). Towards a model of talent development in physical education. Sport. Educ. Soc. 11, 211–230. 10.1080/13573320600813366

[B8] Bentler P. M. (1990). Comparative fit indices in structural models. Psychol. Bull. 28, 97–104. 10.1037/0033-2909.107.2.238 2320703

[B9] Browne M. W. Cudeck R. (1992). Alternative ways of assessing model fit. Sociol. Methods Res. 21, 230–258. 10.1177/0049124192021002005

[B10] Buhaş S. Stance L. (2017). The relationship between personality and physical activity. GeoSport Soc. 2, 72–77.

[B11] Craft A. (2006). Fostering creativity with wisdom. Cambridge J. Educ. 36, 337–350. 10.1080/0305764060086583530532725

[B12] Deci E. L. Ryan R. M. (1985). The general causality orientations scale: self-determination in personality. J. Res. Pers. 19, 109–134. 10.1016/0092-6566(85)90023-6

[B13] Deci E. L. Ryan R. M. (2000). The “what” and “why” of goal pursuits: human needs and the self-determination of behavior. Psychol. Inq. 11, 227–268. 10.1207/S15327965PLI1104_01

[B14] Deci E. L. Ryan R. M. (2004). Handbook of Self-Determination Research. Rochester, NY: University Rochester Press.

[B15] Deci E. L. Ryan R. M. (2008). Facilitating optimal motivation and psychological well-being across life's domains. Can. Psychol. Can. 49, 14–23. 10.1037/0708-5591.49.1.14

[B16] Diener E. Diener M. Diener C. (1995). Factors predicting the subjective well-being of nations. J. Personal. Soc. Psychol. 69, 851–864. 10.1037/0022-3514.69.5.851 7473035

[B17] Eisenberger R. Shanock L. (2003). Rewards, intrinsic motivation, and creativity: a case study of conceptual and methodological isolation. Creat. Res. J. 15, 121–130. 10.1080/10400419.2003.9651404

[B18] Ellis N. Meneely J. (2015). Springboards and barriers to creative risk-taking and resolve in undergraduate interior design studios. J. Inter. Des. 40, 17–40. 10.1111/joid.12065

[B19] Fang R. Liu Y. Sun J. (2012). Exploration and inspection of the conceptual model concerning teenagers' autonomous fitness behavior. China Sport Sci. Technol. 48, 104–116. 10.3969/j.issn.1002-9826.2012.06.017

[B20] Feldman D. H. (1999). “The development of creativity,” in Handbook of Creativity, ed R. J. Sternberg (Cambridge: Cambridge University Press), 169–186.

[B21] Fernández-Abascal E. G. Díaz M. D. M. (2013). Affective induction and creative thinking. Creat. Res. J. 25, 213–221. 10.1080/10400419.2013.783759

[B22] Frith E. Loprinzi P. D. (2018). Experimental effects of acute exercise and music listening on cognitive creativity. Physiol. Behav. 191, 21–28. 10.1016/j.physbeh.2018.03.034 29608999

[B23] Gable P. Harmon-Jones E. (2010b). The motivational dimensional model of affect: Implications for breadth of attention, memory, and cognitive categorisation. Cogn. Emot. 24, 322–337. 10.1080/02699930903378305

[B24] Gable P. A. Harmon-Jones E. (2008). Approach-motivated positive affect reduces breadth of attention. Psychol. Sci. 19, 476–482. 10.1111/j.1467-9280.2008.02112.x 18466409

[B25] Gable P. A. Harmon-Jones E. (2010a). The effect of low versus high approach-motivated positive affect on memory for peripherally versus centrally presented information. Emotion 10, 599. 10.1037/a0018426 20677877

[B26] Gan Q. Bai X. Liu J. Wei R. L M. Xu G.. (2020). Creativity competence: part III of the 5Cs framework for twenty-first century key competences. J. East China Norm. Univ. 38, 57. 10.16382/j.cnki.1000-5560.2020.02.006

[B27] Gerhart B. Fang M. (2015). Pay, intrinsic motivation, extrinsic motivation, performance, and creativity in the workplace: revisiting long-held beliefs. Annu. Rev. Organ. Psychol. Organ. Behav. 2, 489–521. 10.1146/annurev-orgpsych-032414-111418

[B28] Hao N. Tang M. (2017). The effects of motivation on creativity: present situation and prospect. J. East China Norm. Univ. 35, 107–114, 138. 10.16382/j.cnki.1000-5560.2017.04.011

[B29] Hayes A. F. (2017). Introduction to Mediation, Moderation, and Conditional Process Analysis: A Regression-Based Approach. New York, NY: Guilford Publications.

[B30] Hennessey B. A. (2000). Self-determination theory and the social psychology of creativity. Psychol. Inq. 11, 293–298. Available online at: http://www.jstor.org/stable/1449624

[B31] Hennessey B. A. Amabile T. M. (1998). Reality, intrinsic motivation, and creativit. Am. Psychol. 53, 674–675. 10.1037/0003-066X.53.6.6748937264

[B32] Hu L. Bentler P. M. (1999). Cutoff criteria for fit indexes in covariance structure analysis: conventional criteria versus new alternatives. Struct. Equ. Model. a Multidiscip. J. 6, 1–55. 10.1080/10705519909540118

[B33] Huebner E. S. Dew T. (1995). Preliminary validation of the positive and negative affect schedule with adolescents. J. Psychoeduc. Assess. 13, 286–293. 10.1177/07342829950130030732838809

[B34] Ingledew D. K. Markland D. Sheppard K. E. (2004). Personality and self-determination of exercise behaviour. Pers. Individ. Dif. 36, 1921–1932. 10.1016/j.paid.2003.08.021

[B35] Karwowski M. (2012). Did curiosity kill the cat? Relationship between trait curiosity, creative self-efficacy and creative personal identity. Eur. J. Psychol. 8, 547–558. 10.5964/ejop.v8i4.513

[B36] Karwowski M. (2016). The dynamics of creative self-concept: changes and reciprocal relations between creative self-efficacy and creative personal identity. Creat. Res. J. 28, 99–104. 10.1080/10400419.2016.1125254

[B37] Kozbelt A. Beghetto R. A. Runco M. A. (2010). Theories of Creativity. New York, NY: Cambridge University Press.

[B38] Li W. Li X. Huang L. Kong X. Yang W. Wei D.. (2015). Brain structure links trait creativity to openness to experience. Soc. Cogn. Affect. Neurosci. 10, 191–198. 10.1093/scan/nsu041 24603022 PMC4321617

[B39] Lin X. Wang M. (1997). Williams Creativity Assessment Packet. Taibei: Psychology Press.

[B40] Lu J. Liu W. He W. Lu S. (2002). The influence of the students mood on their creativity. Acta Psychol. Sin. 34, 381–386.

[B41] Moneta G. B. Siu C. M. Y. (2002). Trait intrinsic and extrinsic motivations, academic performance, and creativity in Hong Kong college students. J. Coll. Stud. Dev. 45, 664–683. 10.1023/A:1016007529278

[B42] Pessoa L. Engelmann J. B. (2010). Embedding reward signals into perception and cognition. Front. Neurosci. 4, 17. 10.3389/fnins.2010.00017 20859524 PMC2940450

[B43] Prentice M. Jayawickreme E. Fleeson W. (2019). Integrating whole trait theory and self-determination theory. J. Pers. 87, 56–69. 10.1111/jopy.12417 29999534

[B44] Rhodes M. (1961). An analysis of creativity. Phi Delta Kappan 42, 305–310.

[B45] Rhodes R. E. (2006). The built-in environment: the role of personality and physical activity. Exerc. Sport Sci. Rev. 34, 83–88. 10.1249/00003677-200604000-00008 16672806

[B46] Rhodes R. E. Pfaeffli L. A. (2012). Personality and physical activity. Oxford Handb. Exerc. Psychol. 195–223. 10.1093/oxfordhb/9780195394313.013.0011

[B47] Román P. Á. L. Vallejo A. P. Aguayo B. B. (2018). Acute aerobic exercise enhances students' creativity. Creat. Res. J. 30, 310–315. 10.1080/10400419.2018.1488198

[B48] Sansone C. Harackiewicz J. M. (2000). Intrinsic and Extrinsic Motivation: The Search for Optimal Motivation and Performance. San Diego, CA: Academic Press.

[B49] Seymour J. Pratt G. Patterson S. Korman N. Rebar A. Tillston S.. (2021). Changes in self-determined motivation for exercise in people with mental illness participating in a community-based exercise service in Australia. Health Soc. Care Commun. 10.1111/hsc.13588. [Epub ahead of print]. 34614232

[B50] Siemsen E. Roth A. Oliveira P. (2010). Common method bias in regression models with linear, quadratic, and interaction effects. Organ. Res. Methods 13, 456–476. 10.1177/1094428109351241

[B51] Steinberg H. Sykes E. A. Moss T. Lowery S. LeBoutillier N. Dewey A. (1997). Exercise enhances creativity independently of mood. Br. J. Sports Med. 31, 240–245. 10.1136/bjsm.31.3.240 9298561 PMC1332529

[B52] Sutin A. R. Stephan Y. Luchetti M. Artese A. Oshio A. Terracciano A. (2016). The five-factor model of personality and physical inactivity: a meta-analysis of 16 samples. J. Res. Pers. 63, 22–28. 10.1016/j.jrp.2016.05.001 29056783 PMC5650243

[B53] Urban K. K. (1991). On the development of creativity in children. Creat. Res. J. 4, 177–191. 10.1080/10400419109534384

[B54] Wang F. Zheng Y. Bao Y. (2019). Research on the influence mechanism of college students' self - determination on their physical exercise behavior. Bull. Sport Sci. Technol. 9, 149–151. 10.19379/j.cnki.issn.1005-0256.2019.09.063

[B55] Watson D. Clark L. A. Carey G. (1988). Positive and negative affectivity and their relation to anxiety and depressive disorders. J. Abnorm. Psychol. 97, 346. 10.1037/0021-843X.97.3.346 3192830

[B56] Williams F. E. (1980). Creativity Assessment Packet (CAP). New York, NY: DOK Publishers.

[B57] Wilson K. E. Dishman R. K. (2015). Personality and physical activity: a systematic review and meta-analysis. Pers. Individ. Dif. 72, 230–242. 10.1016/j.paid.2014.08.023

[B58] Xia H. Ding Q. Zhuang Y. Chen A. (2018). The brain mechanisms of the physical exercise enhancing cognitive function. Adv. Psychol. Sci. 26, 1857–1868. 10.3724/SP.J.1042.2018.01857

[B59] Xiang M. (2013). The path of promotion on teenagers' physical exercise and health-related well-being:based onself-determination theory model. China Sport Sci. 8, 21–28. 10.3969/j.issn.1000-677X.2013.08.003

[B60] Yan B. Zheng X. Qiu L. (2002). Family income on college students's subjective well-being. Chin. J. Clin. Psychol. 10, 118–119.

[B61] Yu D. Wang J. Tao W. (2021). Management creativity and organizational performance:considering the mediating effect of innovation opportunity identification and the moderating effect of positive emotion. Sci. Technol. Prog. Policy 38, 11–18. 10.6049/kjjbydc.2020070513

[B62] Zhang P. Ding M. Wang C. (2017). The effect of positive emotion experience on creativity based on an empirical study. Stud. Psychol. Behav. 15, 613–618.

[B63] Zhao Y. Qin C. Shu D. Liu D. (2022). Effects of short-term aerobic exercise on creativity. Think. Ski. Creat. 44, 2–10. 10.1016/j.tsc.2022.10103331349179

